# Isolation, Characterization, Differentiation and Immunomodulatory Capacity of Mesenchymal Stromal/Stem Cells from Human Perirenal Adipose Tissue

**DOI:** 10.3390/cells8111346

**Published:** 2019-10-29

**Authors:** Patrick C. Baer, Benjamin Koch, Elena Hickmann, Ralf Schubert, Jindrich Cinatl, Ingeborg A. Hauser, Helmut Geiger

**Affiliations:** 1 Division of Nephrology, Department of Internal Medicine III, University Hospital, Goethe University, 60596 Frankfurt/M., Germany; b.koch@med.uni-frankfurt.de (B.K.); elena.hickmann@gmx.de (E.H.); ingeborg.hauser@kgu.de (I.A.H.); h.geiger@em.uni-frankfurt.de (H.G.); 2 Division of Allergology, Pneumology and Cystic Fibrosis, Department for Children and Adolescents, University Hospital, Goethe University, 60596 Frankfurt/M., Germany; ralf.schubert@kgu.de; 3 Institute of Medical Virology, University Hospital, Goethe University, 60596 Frankfurt/M., Germany; cinatl@em.uni-frankfurt.de

**Keywords:** mesenchymal stromal/stem cells, perirenal, adipose tissue, fat, characterization, stimulation, lipopolysaccharide, cytokines, cytomegalovirus

## Abstract

Mesenchymal stromal/stem cells (MSCs) are immature multipotent cells, which represent a rare population in the perivascular niche within nearly all tissues. The most abundant source to isolate MSCs is adipose tissue. Currently, perirenal adipose tissue is rarely described as the source of MSCs. MSCs were isolated from perirenal adipose tissue (prASCs) from patients undergoing tumor nephrectomies, cultured and characterized by flow cytometry and their differentiation potential into adipocytes, chondrocytes, osteoblasts and epithelial cells. Furthermore, prASCs were stimulated with lipopolysaccharide (LPS), lipoteichoic acid (LTA) or a mixture of cytokines (cytomix). In addition, prASC susceptibility to human cytomegalovirus (HCMV) was investigated. The expression of inflammatory readouts was estimated by qPCR and immunoassay. HCMV infection was analyzed by qPCR and immunostaining. Characterization of cultured prASCs shows the cells meet the criteria of MSCs and prASCs can undergo trilineage differentiation. Cultured prASCs can be induced to differentiate into epithelial cells, shown by cytokeratin 18 expression. Stimulation of prASCs with LPS or cytomix suggests the cells are capable of initiating an inflammation-like response upon stimulation with LPS or cytokines, whereas, LTA did not induce a significant effect on the readouts (ICAM-1, IL-6, TNFα, MCP-1 mRNA and IL-6 protein). HCMV broadly infects prASCs, showing a viral load dependent cytopathological effect (CPE). Our current study summarizes the isolation and culture of prASCs, clearly characterizes the cells, and demonstrates their immunomodulatory potential and high permissiveness for HCMV.

## 1. Introduction

Mesenchymal stromal/stem cells (MSCs) are immature multipotent stromal cells, which represent a rare population in the perivascular niche within fully specialized tissues throughout the whole body [1]. The cells can be isolated from nearly all adult tissues, for example, adipose tissue, solid organs and bone marrow [2,3], and proliferated in vitro. Cultured MSCs release a broad range of growth factors, cytokines, and chemokines [4,5] in the culture supernatant that may improve regeneration in injured cells, organs or tissue. Therefore, MSCs are an optimal source for tissue regeneration support therapies after tissue injury. The organ-protective effects of MSCs, their conditioned medium or extracellular vesicles have been investigated in the last decade, demonstrating that either infused stem cells or their derivates facilitated tissue and organ regeneration predominantly by released regeneration-promoting factors. Furthermore, MSCs have immunological properties, including anti-inflammatory, immunoregulatory and immunosuppressive capacities [6,7], and are, therefore, active immunomodulators during inflammation and sepsis. This immunomodulatory activity has been attributed to the secretion of soluble factors. The MSCs were shown to interact with a variety of immune cells, including CD4 and CD8 T cells, natural killer cells, B cells, monocytes and dendritic cells [8]. The MSCs have also been shown to express Toll-like receptors (TLRs), the major molecules linking innate and adaptive immunity [9]. The TLRs act as sensors for invading pathogens broadly distributed on immune cells and are involved in the pathogenesis of chronic inflammatory and infectious diseases [10].

Recent data have suggested that adipose tissue located in different anatomical locations of the body appear to have distinct cellular compositions and diverse functions [11,12,13]. In humans, fat depot-specific differences are clinically relevant owing to the observation that increased abdominal white fat is associated with insulin resistance, while subcutaneous white adipose tissue exerts a protective effect against metabolic syndrome [11]. Para- and perirenal adipose tissue is a fat pad located in the retroperitoneal space. Perirenal fat is separated from pararenal fat by the renal fascia, and surrounds each kidney [14]. It is a collection of adipose tissue located superficial to the renal cortex and is part of the visceral fat, which can be divided into perirenal, gonadal, epicardial, retroperitoneal, omental and mesenteric fat depots [15]. They are composed mainly of white adipose cells that store energy and produce soluble inflammatory cytokines [14,16]. Perirenal fat shares the same developmental origin as typical visceral fat [14]. However, each white adipose tissue depot can be described as a separate mini-organ [15], and perirenal fat and typical visceral fat are different in histology, physiology and functions [14]. The vascularization of perirenal adipose tissue grows from branches of the abdominal aorta, which also supplies blood to the kidney cortex. Therefore, effects on renal cells through soluble factor released by cells from the perirenal adipose tissue are possible [16]. Renal adipose tissue has been linked recently to effects on kidney function and blood hypertension [17] and a neuronal link from perirenal adipose tissue to multiple central nervous system’s regions has been shown in animal data [18]. Perirenal tissue is rarely analyzed for viral infections, however MSC from selected organs other than perirenal tissue show susceptibility and permissiveness for human cytomegalovirus (HCMV) infection [19,20]

The object of this study was to describe the isolation and culture of human perirenal adipose-derived stromal/stem cells (prASCs) in detail and to characterize cultured cells and their differentiation potential into adipocytes, chondrocytes, osteoblasts and epithelial cells. The present study further investigated the immunomodulatory potential of prASCs after stimulation with lipopolysaccharide (LPS), lipoteichoic acid (LTA), a mixture of cytokines (cytomix), or infection with HCMV. Whereas, few studies used human prASCs in vitro [21,22,23], there is currently no other study which fully described the isolation, characterization, differentiation and immunomodulatory potential of human prASCs as well as their susceptibility to HCMV.

## 2. Materials and Methods

### 2.1. Perirenal Adipose Tissue

Human perirenal adipose tissue was obtained from patients undergoing tumor nephrectomies. This study was approved by the ethics committee of the clinic of the Goethe University, Frankfurt (UGO 03/10, Amendment).

### 2.2. Cell Isolation and Culture

Human MSCs were isolated from perirenal adipose tissue from 15 different donors. The tissue was minced by using two scalpels and disintegrated as small as possible for cell isolation. The minced tissue was then digested at 37 °C with collagenase (1 mg/mL; CellSystems, Troisdorf, Germany) and continuous agitation for 60 min. Cells were then separated from the remaining fibrous material and the floating adipocytes by centrifugation at 300× *g*. The pelleted cells were collected, and the procedure was repeated twice. The sedimented cells were washed with phosphate-buffered saline (PBS) and filtered through a 125-µm plastic mesh (Millipore, Schwalbach, Germany). Erythrocyte contamination, if required, was reduced by density gradient centrifugation with Bicoll (Biochrom, Berlin, Germany), because high erythrocyte contamination was found to decrease ASCs adherence and proliferation markedly. It was observed in previous experiments, that a preceding density gradient separation provided a better yield of adipose-derived stromal/stem cells (ASCs) than treatment with an erythrocyte lysing buffer [24]. Finally, the cells were plated for initial cell culture and cultured at 37 °C in an atmosphere of 5% CO_2_ in humid air. Dulbecco’s modified Eagle’s medium (DMEM; Sigma, Taufkirchen, Germany) was used with a physiologic glucose concentration (100 mg/dl) supplemented with 10% fetal bovine serum (FBS; Biochrom, Berlin, Germany) as the standard culture medium. Primary isolated cells were intensively washed with PBS after 18–24 h of initial plating to remove debris and non-adherent cells. The medium was then replaced every three to four days. Subconfluent cells were passaged by trypsinization. In all experiments we used cultured prASCs in early passages (between 2 and 5).

### 2.3. Cell Characterizations Using Flow Cytometry and Immunofluorescence Staining

Cell morphology was examined by phase contrast microscopy. Flow cytometric analysis was used to show the characteristic marker expression of cultured prASCs. Cells were detached from the cell culture plastic and stained with directly labeled antibodies (CD73-PerCP-eFluo710 (eBioscience, San Diego, CA, USA), CD90-FITC (BD Bioscience, Heidelberg, Germany), CD105-PE, CD29-FITC and CD45-PE (all from Immunotools, Friesoythe, Germany)). The labeled prASCs were then measured using a flow cytometer (BD Biosciences, Heidelberg, Germany). All experiments included negative controls with corresponding isotype controls. Cells were gated by forward and sideward scatter to eliminate debris.

Cells were cultured on chamber slides (Nunc Lab-Tech^®^), rinsed three times with PBS and fixed with ice-cold methanol/acetone (1:1) for 5 min for immunofluorescence staining. Unspecific binding sites were blocked with PBS containing 5% normal goat serum for 20 min. Primary antibody (anti-vimentin or anti-cytokeratin 18 (CK-18); both from ExBio, Vestec, Czech Republic) was applied after washing and incubated for 45 min at 37 °C with gentle shaking. Afterwards, cells were washed and incubated with a Cy^3^-conjugated secondary monoclonal antibody for 45 min at 37 °C. All dilutions of antibodies were made in PBS containing 1% goat serum. 4,6-diamidino-2-phenylindole dihydrochloride (DAPI; 0.5 µg/mL) was added to the secondary antibody solution for nuclear staining. Controls of nonspecific fluorescence were performed on fixed cells processed without the primary antibody. The cells were washed and covered in Moviol. The slides were stored at 4 °C and analyzed with a fluorescence microscope (Zeiss, Heidelberg, Germany).

### 2.4. Induction of Cell Differentiation

The trilineage differentiation potential of cultured prASCs was induced by incubation in differentiation media for 14 days, followed by the verification of differentiation by standard staining methods (Oil Red O, Alcian Blue, and Alizarin staining, respectively), as further described. Media were changed every three to four days.

Adipogenic differentiation was induced in adipogenic medium containing high glucose content (4.5 g/L), insulin (1.74 µM, Novo Nordisk), dexamethasone (0.1 µM, Ratiopharm), isobutyl-methylxanthin (0.5 mM, Sigma), indomethacine (200 µM, Fluka), and 10% FBS. Oil Red O (Sigma) staining revealed the accumulation of lipid droplets in intracellular vacuoles indicating adipogenic differentiation.

The chondrogenic differentiation of prASCs was induced in chondrogenic medium containing ascorbic acid (50 nM; Merck), insulin (6.25 µg/mL, Novo Nordisk), transforming growth factor β (10 ng/mL, Peprotech) and 1% FBS. The chondrogenic phenotype was assessed by Alcian Blue 8GX staining (Fluka).

Osteogenic differentiation of prASCs was induced in osteogenic medium containing ascorbic acid (50 µM; Merck), glycerophosphate (10 mM, Sigma), dexamethasone (1 µM, Ratiopharm), recombinant bone morphogenic protein-2 (100 ng/mL, Immunotools, Friesoythe) and 15% FBS. After 14 days of incubation, the osteogenic phenotype was assessed by staining according to Alizarin Red S staining (Fluka).

The cells were incubated with all-trans retinoid acid (ATRA; Sigma) at a final concentration of 5 µM for epithelial differentiation. This concentration was determined by taking a pattern of our previous studies and testing with proliferation and vitality assays [25,26]. A stock solution of ATRA dissolved in dimethyl sulfoxide at 10 mM was kept at −80 °C. The ATRA was dissolved in DMEM substituted with 10% FBS for cell culture. The equivalent volume of solvent (dimethyl sulfoxide) without ATRA was used in control samples. Epithelial differentiation medium was replaced every three to four days during a total incubation period of 14 days, followed by analysis of the epithelial differentiation by expression of CK-18 using qPCR, Western blotting and immunofluorescence staining

### 2.5. Stimulation with LPS, LTA, and Cytokines

Cells were grown in 24-well culture plates (for IL-6 measurements in the supernatant) or small cell culture flasks (25 cm^2^ for PCR analyses) to subconfluence. Cells were then washed with PBS and treated with TLR-4 ligand LPS (LPS-EB ultrapure from E. coli 0111: B4; 10, 100, 1000 ng/mL; Invivogen, San Diego, USA, Cat. No. tlrl-3pelps), TLR-2 ligand LTA (from Staphylococcus aureus; 1000 ng/mL; Invivogen, Cat. No. tlrl-slta), or cytomix (IFNγ, 200 U/mL; IL-1β, 25 U/mL, and TNFα, 10 ng/mL) diluted in DMEM with 10% FBS. The RNA was isolated after 4 h of stimulation, and supernatants were harvested after 48 h for IL-6 quantification. Therefore, supernatants were collected, centrifuged at 300× *g* for 5 min and assessed for the cytokine by an immunoassay or stored at −20 °C for later measurement.

### 2.6. HCMV Infection

prASCs were infected with HCMV patient isolate Hi91 [27] at a multiplicity of infection (MOI) of 0.05, 0.5, 1 and 4. Expression of HCMV-specific late antigen was detected 96 h post-infection by immunoperoxidase staining using monoclonal antibodies directed against gB/gpUL55-encoded antigen (kindly provided by K. Radsak, Institut für Virologie, Marburg, Germany) as previously described [28]. Other samples were used for extraction of total RNA and cDNA synthesis. Changes in gene expression of selected targets were quantified by qPCR in triplicate measurements.

### 2.7. Cell Viability Assays

Cell viability of prASCs was determined by by two viability assays, a photometric assay using 2,3-Bis-(2-Methoxy-4-Nitro-5-Sulfophenyl)-2*H*-Tetrazolium-5-Carboxanilide (XTT), as described previously [29], and a fluorescent-based assay using calcein-acetoxymethyl (calcein-AM, Biolegend, San Diego, USA), to determine any possible cytotoxic effects during the stimulations. In brief, 5000 cells/well were seeded in 96-well plates. We measured each stimulation in quintuplicate for each biological replicate. One day after seeding, the prASCs were stimulated for 96 h as described above. The XTT reagent was then added to the wells, as described by the manufacturer (AppliChem, Darmstadt, Germany), and incubated at 37 °C for 4 h. Absorbance was measured in an Apollo LB911 microplate reader (Berthold, Bad Wildbad, Germany) at 492 vs. 650 nm. Data are expressed as arbitrary units and calculated as a percent in relation to the control. For the fluorescence assay, cells were washed and calcein-AM (1 µM) was added incubated at 37 °C for 30 min. Then, fluorescence was measured immediately using a fluorescence reader (BMG Fluostar, Ortenberg, Germany) with excitation and emission wavelengths of 485 and 515 nm. Cells incubated in buffer without calcein-AM were used as background controls. Data are expressed as arbitrary fluorescence units.

### 2.8. PCR

We performed a single-step RNA isolation protocol using Nucleozol (Macherey-Nagel, Düren, Germany) to extract RNA from cultured prASCs. Then, cDNAs were synthesized from isolated RNA for 30 min at 37 °C using 1 µg RNA, 50 µM random hexamers, 1 mM deoxynucleotide-tripheosphate mix, 50 units of reverse transcriptase (Fermentas, St. Leon-Rot, Germany) in 10× PCR buffer, 1 mM β-mercaptoethanol and 5 mM MgCl_2_. A Hot FIREPol EvaGreen Mix Plus was used (Solis Biodyne, Tartu, Estonia) for the master mix; the primer mix and RNAse-free water were added. Quantitative PCR (qPCR) was carried out in 96-well plates using the following conditions: Twelve minutes at 95 °C for enzyme activation, 15 s at 95 °C for denaturation, 20 s at 63 °C for annealing and 30 s at 72 °C for elongation (40 cycles). Finally, a melting curve analysis was executed. The quantification of the PCR fragment was performed using the ABI Prism^®^ 7900HT Fast Real-Time PCR System with a Sequence Detection System SDS 2.4.1 (Thermo Fisher Scientific). Relative quantification was assessed by the ∆∆CT method [30], using β-actin as a calibrator, and levels of target gene expression were estimated by 2^−∆∆*C*t^. In selected experiments, PCR products were separated by agarose gel electrophoresis (2%) and observed under UV illumination. Primer pairs were synthesized by Thermo Fisher Scientific (Germany) and are listed in Table 1.

### 2.9. Western Blot

The cells were processed for Western blotting, as described previously [31]. In brief, the cells were lysed using 10 mM Tris pH 7.4, 0.1% SDS, 0.1% Tween20, 0.5% TritonX100, 150 mM NaCl, 10 mM EDTA, 1 M urea, 10 mM NEM, 4 mM benzamidine and 1 mM PMSF and collected by scraping. After centrifugation, the pellet was suspended in Laemmli’s buffer and heated at 95 °C for 5 min prior to electrophoresis on a 10% SDS polyacrylamide gel. The protein content was determined by a standard assay and an equal volume of protein was loaded into each lane. The separated proteins were transferred electrophoretically to Immobilon transfer membrane (Millipore). Membranes were blocked for 2 h. Immunoblotting was performed by incubating with antibodies against CK-18 (resulting in a 45 kDa band, ExBio, Vestec, Czech Republic) or β-Actin (resulting in a 42 kDa band, Sigma Aldrich), followed by a secondary antibody (horseradish peroxidase-conjugated anti-mouse or anti-rabbit IgG; Amersham Pharmacia). Protein bands were made visible using the Peqlab Fusion FX system (VWR, Darmstadt, Germany) followed by densitometric evaluation using ImageJ 1.8.0 (NIH, www.nih.gov).

### 2.10. Immunoassay

Interleukin-6 was quantified using a commercially available enzyme-linked immunosorbent assay kit (ELISA) (Immunotools, Friesoythe, Germany). In brief, the wells of 96-well microtiter plates were coated with an anti-human IL-6 antibody overnight at room temperature (RT). Nonspecific binding sites were blocked with PBS/2% BSA/0.05% Tween20 for 1 h. The plates were then washed with PBS/0.05% Tween and the standard (8–500 pg/mL), and the samples were added for 2 h at RT. All samples were diluted in assay buffer (1:25) and run in duplicate. The plates were washed and incubated with biotinylated anti-IL-6 for 2 h at RT, washed again and incubated with horseradish-peroxidasestreptavidin for 30 min. After washing, TMB was added for 5–20 min and the substrate reaction was stopped and measured (450 vs. 620 nm). The data are presented as ng/*mL* of IL-6 in the supernatant.

### 2.11. Statistical Analysis

The data are expressed as mean ± standard deviation (SD). Analysis of variance with Dunnett’s Multiple Comparison Test or Student’s t-test were used for statistical analysis. *p* values < 0.05 were considered significant.

## 3. Results

### 3.1. Isolation and Characterization of prASCs

We used an average of 75 g of perirenal adipose tissue to isolate prASCs, yielding 6.9 × 10^8^ cells seeded in total, corresponding to approximately 9.2 × 10^6^ primary isolated cells per gram tissue. Nevertheless, only some of these cells adhere to cell culture plastic and proliferate. Approximately 80–90% of the isolated cells do not adhere and were aspirated with the first washing after 24 h. Adhered primary cells cultured in a 75 cm^2^ cell culture flask need up to seven days to reach subconfluence (~80–85%), the situation where the cells were subcultured for the first time. At this time, an average of 3.75 × 10^5^ cells were grown in the 75 cm^2^ cell culture flask (corresponding to 5000 cells/cm^2^ growth area).

**Figure 1:**
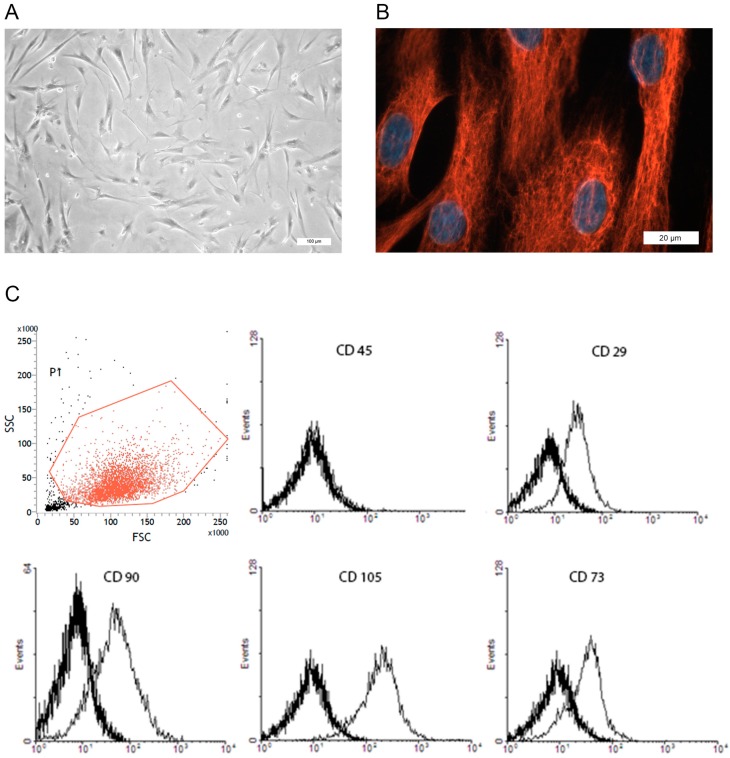
Characterization of human perirenal mesenchymal stromal/stem cells (prASCs) in vitro. (**A**) Characteristic phase contrast microscopy of prASCs in passage 2 (bar: 100 µm); (**B**) Immunofluorescence staining of intermediate filament vimentin, nuclei were counterstained with DAPI (bar: 20 µm); (**C**) Representative flow cytometric overlay histograms of characteristic marker expression (CD73, CD90, CD105, CD29) and of CD45, a pan leukocyte marker which is not expressed on MSCs. Thick black histograms represent isotype controls. A dot plot shows the forward and sideward scatter analysis with the gating strategy to eliminate debris.

Cultured prASCs displayed a spindle-shaped fibroblastoid morphology (Figure 1A). Primary isolated cells are morphologically more heterogeneous than cultures after passaging. Nevertheless, cultured cells became morphologically increasingly homogeneous in higher passages. Contaminations with cells of epithelial morphology or pre-adipocytes were not detectable in the culture at passage 2. In addition, immunofluorescence staining in passage 2 revealed that all the cells cultured (100%) expressed vimentin (Figure 1B), also showing a very homogeneous cell culture of mesenchymal origin. There were no vimentin-negative cells detectable in any staining done.

The cells were also characterized by flow cytometric analysis utilizing characteristic markers for MSCs in vitro. Cultured prASCs expressed CD29, CD73, CD 90 and CD105 but did not express CD45 (Figure 1C). Furthermore, cultured prASCs were positive for CD44 and CD166 and did not express the endothelial markers CD31 and C11b, which are expressed on the surface of many leukocytes, including monocytes, granulocytes and macrophages (data not shown).

### 3.2. Differentiation of prASCs

**Figure 2:**
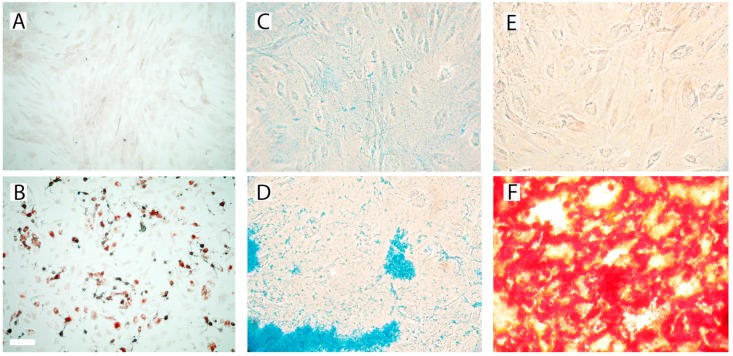
Trilineage differentiation of cultured prASCs. Differentiation into adipocytes, chondrocytes and osteoblasts was induced by adipogenic (**B**), chondrogenic (**D**) and osteogenic (**F**) medium for 14 days. Control cells were cultured in standard culture medium for 14 days (**A**,**C**,**E**) After 14 days of incubation in either standard or differentiation medium, cultures were stained with Oil Red O (**A**,**B**), Alcian Blue 8GX (**C**,**D**) or Alizarin Red S (**E**,**F**) (bar: 100 µm).

We investigated the differentiation potential of prASCs into adipocytes, chondrocytes, osteoblasts and epithelial cells. We used the differentiation media described in the case of trilineage differentiation of cultured prASCs. After 14 days of incubation, the verification of differentiation was done by standard staining methods (Oil Red O, Alcian Blue and Alizarin staining, respectively). Incubation of undifferentiated prASCs for 14 days under adipogenic conditions induced the de novo formation of cytoplasmatic lipid droplets, a characteristic of pre-adipocytes, stained by Oil Red O staining (Figure 2B). Chondrogenic-induced prASCs exhibit an intense blue color following Alcian Blue 8GX staining (Figure 2D), indicative of cartilage extracellular matrix accumulation. Induction of osteogenic differentiation of the cells for 14 days resulted in the deposition of mineralized nodules that stained red by Alizarin Red S staining (Figure 2F), characteristic for osteoblasts. Cells cultured in osteogenic induction medium changed from an elongated mesenchymal appearance to a multilateral form with a tightly packed multilayer.

Control cells cultured in standard medium were not stained by Oil Red O (Figure 2A), Alcian Blue 8GX (Figure 2C) or Alizarin Red staining (Figure 2E).

**Figure 3:**
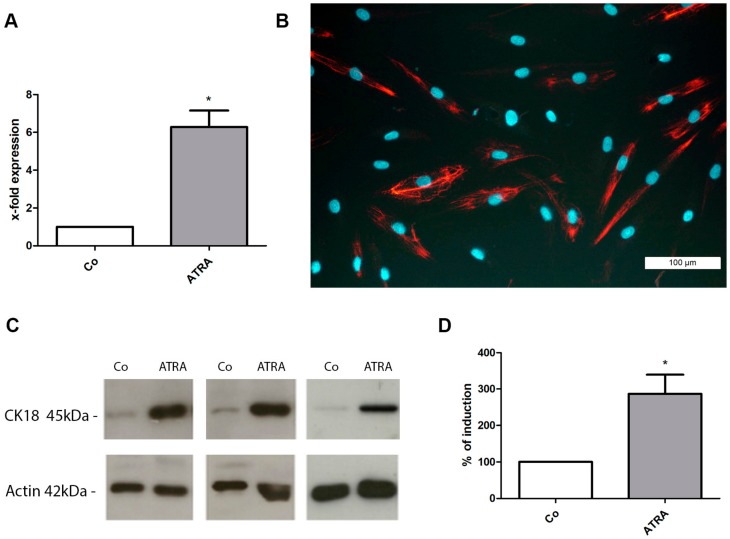
Induction of epithelial differentiation. (**A**) Analysis of cytokeratin 18 (CK-18) induction by qPCR. The expression levels in each experiment were normalized using β-actin as a housekeeping gene and are expressed relative to the unstimulated control using the ∆∆CT method (n = 6; * *p* < 0.05); (**B**) Characteristic immunofluorescence staining of CK-18 after epithelial differentiation with ATRA (14 days). Nuclei were stained with DAPI (bar: 100 µm); (**C**) Characteristic Western blots. Expression of CK-18 (45 kDa) and β-actin (42 kDa) of the unstimulated control cells (Co) and after incubation with ATRA (5 µM) for 14 days; (**D**) Densitometric evaluation of CK-18 induction in relation to undifferentiated controls (= 100%) (*n* = 5; * *p* < 0.05).

In addition to their trilineage differentiation potential, prASCs were also able to differentiate into the epithelial lineage. In order to elucidate the influence of ATRA on prASCs differentiation, expression of CK-18, an early epithelial marker, was evaluated by qPCR analysis (Figure 3A), immunofluorescence staining (Figure 3B) and Western blotting (Figure 3C,D). After incubation with ATRA for 14 days, the expression of CK-18 mRNA was 6.2-fold induced compared to unstimulated control cells (Figure 3A). Immunofluorescence staining revealed that approximately 45% of the cells were CK-18 positive (Figure 3B), and Western blot analysis also clearly showed the significant induction of CK-18 protein in epithelial-induced prASCs (Figure 3C) (densitometric analysis calculated in percent: control (standard medium) = 100% (background signal); ATRA = 286% (Figure 3D)).

### 3.3. Stimulation with LPS, LTA and Cytokines

**Figure 4:**
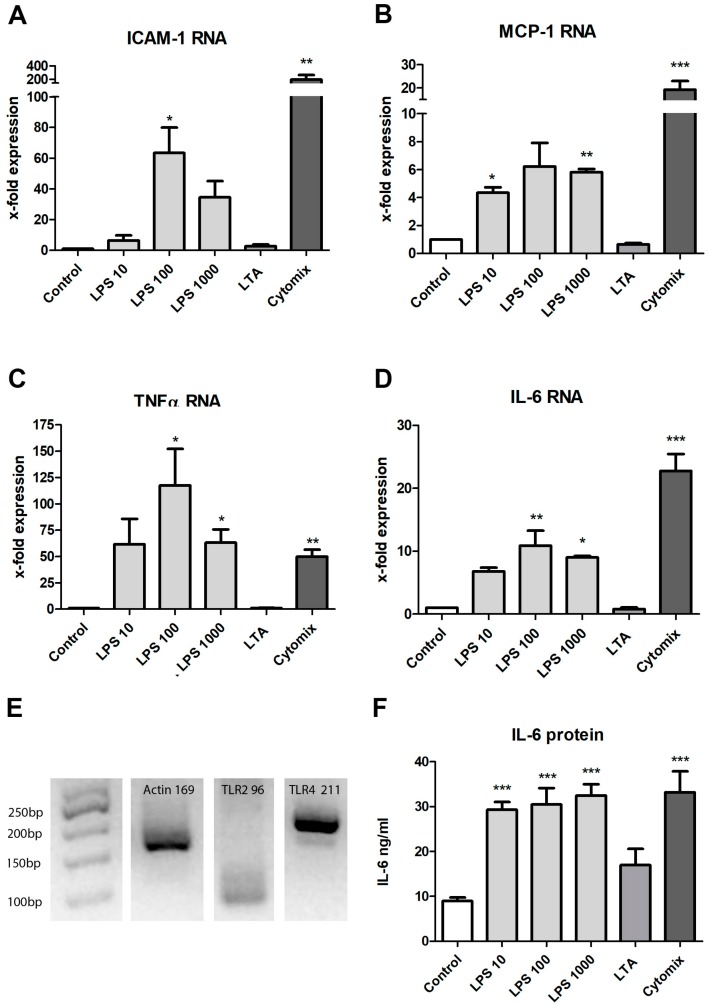
Effect of lipopolysaccharide (LPS) (10, 100, 1000 ng/mL), LTA (1000 ng/mL) or a cytokine mix (cytomix). (**A**–**D**) Assessment at the mRNA level by qPCR for ICAM-1, MCP-1, IL-6, and TNFα after 4 h stimulation. The expression levels in each experiment were normalized to a housekeeping gene (β-actin) and are expressed relative to the control using the ∆∆CT method. (mean ± SD; * *p* < 0.05, ** *p* < 0.01, *** *p* < 0.001 versus control), *n* = 4.; (**E**) Constitutive expression of TLR-2- and TLR-4-mRNA of prASCs in vitro. The PCR products were separated by agarose electrophoresis and observed under UV illumination (TLR-2: 96 kb, TLR-4: 211 kb, and β-actin: 169 kb (housekeeper)); (**F**) Quantification of IL-6 protein in the supernatant after 48 h stimulation. Data from four independent immunoassays (ELISA) are represented as mean ± SD (*** *p <* 0.0001).

We added increasing concentrations of LPS, a major component of the outer membrane of Gram-negative bacteria, to the cell supernatant and evaluated the response by qPCR and an immunoassay to test the responsiveness of prASCs to pro-inflammatory stimuli. In addition, we stimulated the cells with LTA, a key cell wall component of Gram-positive bacteria and potent stimulator of TLR-2. A mixture of pro-inflammatory cytokines (cytomix: IFNγ, 200 U/mL; IL-1β, 25 U/mL; and TNFα, 10 ng/mL) was used as a positive stimulation control. In particular, prASCs produced a set of inflammatory mediators in response to the bacterial or cytokine stimulation. The mRNA expression of ICAM-1, MCP-1, TNFα and IL-6 was upregulated in response to the TLR-4 agonist LPS (Figure 4A–D). The cytomix induced a significantly higher induction of ICAM-1, MCP-1 and IL-6 than stimulation with LPS, whereas there were no significant differences in the induction of TNFα mRNA expression. We also detected no significant differences between the different stimulation doses of LPS. On the other hand, LTA induced no significant effect on the cytokines analyzed, whereas TLR-2 was shown to be expressed (Figure 4E). Total mRNA was isolated from the prASCs in passage 2 cultured in standard medium to determine the constitutive expression of the relevant receptors for the bacterial infections (TLR-2 for LTA and TLR-4 for LPS). Both receptors were found to be expressed, whereas TLR-2 only showed a weak but specific signal at 96 bp.

Furthermore, qPCR analysis revealed that some mRNAs were constitutively expressed in vitro. IL-6 and MCP-1 mRNA were highly detectable in the unstimulated control (CT values approximately 22 and 28, respectively (water: undetectable)), and ICAM-1 was slightly expressed compared with the unstimulated control (CT value approximately 30 (water: 37)). On the other hand, TNFα mRNA was not detected in the unstimulated prASCs.

At the protein level, IL-6 is constitutively released by prASCs (8.9 ± 1.5 ng/mL; mean ± SD, *n* = 4). When the cells were stimulated by LPS or exposed to an inflammatory environment by the cytomix, the production of IL-6 was significantly upregulated (Figure 4F). Nevertheless, we found no dose-dependent effect of the LPS stimulation, and the release of LPS- and cytokine-stimulated IL-6 protein was nearly comparable (LPS 10: 29.3 ± 3.3; LPS 100: 30.5 ± 7.2; LPS 1000: 32.5 ± 4.9; cytomix 33.5 ± 9.3 ng/mL, mean ± SD, *n* = 4)). The LTA also induced an increase in IL-6 release, but the effect was not significant (17.0 ± 7.1 ng/mL; mean ± SD, *n* = 4) (Figure 4F).

These results suggest that, under these culture conditions, prASCs are capable of initiating an inflammation-like response upon stimulation with LPS or cytokines. Interestingly, only LPS (from gram negative bacteria) induced a significant stimulation of prASCs, but no significant stimulation was found by after incubation with the pathogen of gram-positive bacteria.

**Figure 5:**
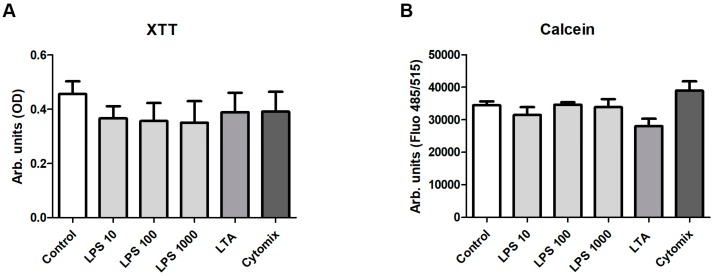
Cell viability after stimulation with LPS, LTA or cytomix for 96 h. A total of 5000 cells were cultured in 96 well plates (*n* = 3, each in quintuplicate) and stimulated for 96 h. (**A**) The XTT assay was performed and optical density (OD) was measured in a microplate reader at 492 vs. 650 nm (arbitrary units). (**B**) The calcein assay was performed and fluorescence was measured in a reader at 485 nm (excitation) and 515 nm (emission). No significant effects of the different stimulations could be detected with both assays (mean ± SD, *n* = 3, each in quintuplicate).

To investigate the effects of LPS, LTA and the cytomix on prASCs viability, we used two viability assays using XTT or calcein-AM. The XTT assay is a colorimetric assay used to determine viability based on the metabolic activity of the cultured cells. Calcein-AM is a cell permeant non-fluorescent dye, which is in live cells intracellularly converted into calcein, a dye with intense green fluorescence. Whereas a slightly reduced metabolic activity after each stimulation regimen could be detected with the XTT assay (XTT calculated in percent: control, 100%; LPS 10, 80.02%; LPS 100, 76.86%; LPS 1000, 74.86%; LTA, 83.91%; cytomix, 84.24%), the differences in live cell detection were very low. No statistically significant difference could be found, either with the XTT assay (Figure 5A) or with the calcein assay (Figure 5B).

### 3.4. Infection with HCMV

**Figure 6:**
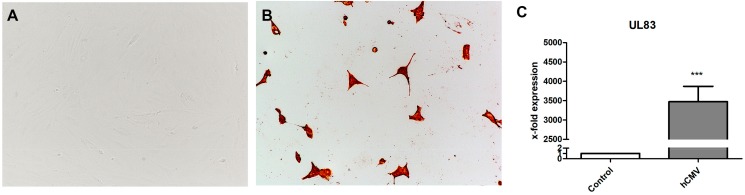
Infection of prASCs with HCMV Hi 91 for 96h. (**A**, **B**) Bright field microscopy of mock-infected prASCs (**A**) and HCMV-infected cells (**B**). Compared to the mock-infected controls (**A**) a strong CPE with very few adherent cells is seen 96 h after infection with HCMV at a MOI of 4 (**B**). All remaining cells are positive for HCMV late antigen, as shown by immunostaining. (**C**) Levels of UL83 mRNA were assessed after 96h. The expression levels in each experiment were normalized to β-actin and are calculated relative to the control using the ∆∆CT method (mean ± SD; * *p* < 0.05 versus control, *n* = 3).

Experiments with prASCs infection were done by HCMV strain Hi91, using a MOI of 0.05, 0.5, 1 and 4. Our results showed prASCs to be susceptible to HCMV even at MOI 0.05. The increase of viral load resulted in an augmented number of infected prASCs and progressive CPE (Figure 6B). Only very few cells were still adherent at 96 h post infection (MOI of 4), all being highly positive for HCMV gB/late antigen (Figure 6B). The HCMV specific mRNA of UL83-coded phosphoprotein HCMV virion protein was absent in controls and strongly expressed in prASCs 96 h after infection (Figure 6C), thus showing permissiveness of the cells.

## 4. Discussion

The abundance of MSCs, their multipotency and ability to secrete various cytokines, and their immunomodulatory effects account for their key role in current tissue regeneration approaches (i.e., Regenerative Medicine) [6]. The MSCs are present in all organs and tissues in vivo [2], and it has also been demonstrated that adipose tissue is a rich source of MSCs. Recent studies have explored the isolation, culture and characterization of MSCs from different adipose tissue depots [32]. The differences in gene expression between subcutaneous and visceral adipose tissue depots—when looking at total fat—have been described [32]. Regarding the tissue source, attention has also been paid to differences between cells derived from subcutaneous and visceral adipose tissue, and emerging evidence shows that there is also a significant variation between different visceral depots [32,33]. There is clear evidence that distinct differences in the expression profiles of developmental genes exist between all adipose tissue depots [32]. Nevertheless, we have not compared the ASCs isolated from different adipose origin in our current study. Adipose tissues have generally been classified as loose connective tissues [14]. Perirenal fat shares the same developmental origin as typical visceral fat. However, the perirenal adipose tissue is an atypical visceral fat pad with a complete system of blood supply, lymph fluid drainage and innervation [14] with near proximity to the kidneys. Perirenal adipose tissue is rarely described as the source of MSCs (or ASCs in particular). One possible reason for this is that the perirenal tissue cannot be easily recovered and an intervention in the perirenal space must be indicated for the removal. Our current study was conducted to characterize cultured MSCs from this cell source and to prove the potential of prASCs. The possible clinical use of prASCs for regenerative therapies is clearly restricted due to the limited tissue source. Thus, liposuction aspirate is easier to access as a cell source and, thus, better explored. In this study, we described the isolation and culture of human prASCs in detail, characterized cultured cells, investigated their multipotential differentiation, including epithelial differentiation, and their immunomodulatory capabilities in response to inflammatory stimuli. The isolation of prASCs could be done according to a protocol from liposuction aspirate established already [25,26]. Perirenal fat is another suitable source of ASC isolation, as also shown by others [21,22]. However, not all of our isolations could be successfully cultured. The cells were from morbid donors who had to be nephrectomized due to a malignant disease. Variability in the tumor dignity could be an explanation, in addition to genetic factors, for the inadequate growth of some isolations. A comparison of MSCs from different tissues, however, showed that MSCs from perirenal fat tissue have the same morphology and phenotypic characteristics as MSCs from other sources.

Since the first description of ASCs and their trilineage differentiation potential by Zuk and co-workers in 2001, many other lineages have been explored where ASCs can differentiate and how this differentiation can be induced. Several studies have shown that ASCs are able to differentiate into epithelial cells when cultured in media containing a retinoid (ATRA) [26], conditioned medium from epithelial cells [34] or a breast cancer cell line [35]. ATRA is an active metabolite of vitamin A and belongs to the retinoids. Retinoids have important functions in the growth and differentiation of tissues in vertebrates. An earlier study from our group showed the de novo expression of CK-18 and a reduction expression of the mesenchymal intermediate filament vimentin [26]. The type I intermediate filament CK-18 is characteristic of cells of single-layered epithelia and essential for normal tissue structure and function. Thus, CK-18 forms an important marker for the identification of epithelial cells. This result is considered to be the first step towards epithelial differentiation, because the intermediate filament vimentin is expressed exclusively in cells of mesenchymal origin. In the present work, medium containing ATRA was also used to stimulate the epithelial differentiation of prASCs. After two weeks of culture in induction media, we verified the epithelial differentiation by the induced expression of the characteristic marker CK-18.

The ASCs were also shown to play a role in the control of tissue inflammation and immunomodulation. The MSCs adopt an immunoregulatory phenotype in response to incubation (or pre-conditioning) with inflammatory factors (e.g., γ-Interferon (IFNγ), Interleukin 1β (IL-1β) or Tumor Necrosis Factor-a (TNFα)), in vivo secreted by activated immune cells [36]. In this context, administration of (pre-conditioned) ASCs could be used to decrease the severity of inflammation. Exemplarily, pre-conditioning of ASCs with inflammatory cytokines prior to cell transplantation is considered as a way to boost their immune regulatory function [37]. IFNγ, a pro-inflammatory cytokine acting against viral and bacterial infections, is one representative source for a pre-conditioning regimen for functional enhancement and upregulation of (pro- and anti-) inflammatory mediators [38,39]. The ASCs incubated with TNFα increased the secretion of IL-6 and IL-8, resulting in promoting endothelial progenitor cell homing and stimulating angiogenesis in an ischemic hindlimb model [40]. The ASCs pre-conditioned with IFNγ, TNFα and IL-6 showed enhanced immunosuppressive properties in vitro [41]. MSCs have also been shown to execute an immunosuppressive effect on lymphocyte proliferation in vitro [42]. In this context,, the inhibitory effect of histocompatibility locus antigen (HLA)—G, expressed and released by MSCs, has been shown [43]. Nevertheless, we did not evaluate the effects of stimulated prASCs on lymphocyte populations and also have not determined the HLA-G expression of prASCs. In the current study, we used a mixture of three cytokines to investigate the stimulatory response of prASCs and measured the induction of pro-inflammatory cytokines (IL-6 and TNFα), a chemokine (MCP-1) and a cell adhesion molecule (ICAM-1). Taken together, pre-conditioning with inflammatory cytokines is a potential way to improve the therapeutic effectiveness for tissue injury and inflammatory disease [37,38].

In addition, ASCs also get in contact with bacterial components during invasive infections. Not much is known about how these pathogens (i.e., LPS and LTA) interact with ASCs and how contact to bacteria influences the secretome in ASCs. The TLRs mediate the activation process of cells by recognizing pathogen-associated molecular patterns, such as LPS or LTA. Activation of TLRs promotes the expression of various inflammatory cytokines, such as TNFα and other costimulatory molecules, and, therefore, initiates adaptive immune responses. Nevertheless, we were not able to show a significant stimulation via TLR-2 induced by LTA. Whereas most reports describe the anti-inflammatory properties (or immune suppressive) of MSCs, other reports target the pro-inflammatory characteristics of pre-conditioned MSCs. Studies reported that the activation of TLR-4 via LPS turned MSCs into a pro-inflammatory phenotype, whereas TLR-3 activation modified MSCs into an anti-inflammatory phenotype [38,44]. Incubation with low-dose LPS limited the immunosuppressive effects of ASCs by increasing IL-6 and TNFα expression, and also some growth factors [45]. Furthermore, it has been demonstrated that proliferation and osteogenic differentiation of adipose-derived MSCs is influenced by LPS [46,47]. Another study showed no significant influence of LPS or LTA on the migration rate and chemotaxis of MSCs [48]. More in vivo studies are needed to understand the immunomodulatory mechanisms of MSCs and the enhancement of this potential by in vitro pre-conditioning regimens.

The susceptibility and permissibility of prASCs to HCMV was not known before our experiments, but is consistent with reported productive infection of MSC derived from bone marrow [20], umbilical cord Wharton’s jelly [49], placenta [50] and MSC from subcutaneous adipose tissue [51] The perivascular niche was described as a relevant MSC origin across the human organs [1]. In addition perivascular stromal cells host HCMV and are a likely long term reservoir [52]. Cell susceptibility to HCMV depends on entry receptor expression, like e.g., b3-Integrin (CD61), which is expressed on ASC [53] and the expression pattern of b1-integrin ((CD29) [54] which is also present on prASCs (see Figure 1C). Pathophysiologically, HCMV can induce an impairment of bone marrow-derived MSCs effector functions [55] and MSCs’ differentiation potential [51]. Because of the perivascular cells’ impact on vascular health further studies elucidating the in vitro and in vivo effects of HCMV infection in prASCs are warranted.

In summary, this study is the first comprehensive description of MSCs from perirenal adipose tissue in vitro. Our current study describes the isolation and culture of human prASCs, characterized cultured cells and the differentiation potential of prASCs into adipocytes, chondrocytes, osteoblasts and, for the first time, into epithelial cells. Furthermore, we showed the immunomodulatory potential of prASCs after stimulation with LPS or cytomix, and—also for the first time—high susceptibility as well as permissivity to HCMV. Interestingly, only LPS (from gram negative bacteria) induced a significant stimulation of prASCs, but no significant stimulation was found by after incubation with the pathogen of gram-positive bacteria.

## Figures and Tables

**Table 1:** Primer used for qPCR analyses.

Gene	Primer Forward	Primer Reverse	Product Length (bp)	NCBI Reference Sequence
*CK-18*	CAC AGT CTG CTG AGG TTG GA	CAA GCT GGC CTT CAG ATT TC	110	NM_000224
*ICAM-1*	CAGTGACTGTCACTCGAGATCT	CCTCTTGGCTTAGTCATGTGAC	500	NM_000201.3
*IL-6*	AAAGATGGCTGAAAAAGATGGATGC	ACAGCTCTGGCTTGTTCCTCACTAC	150	NM_000600.4
*MCP-1*	CCCCAGTCACCTGCTGTTAT	AGATCTCCTTGGCCACAATG	135	NM_002982.4
*TNFα*	CGGGACGTGGAGCTGGCCGAGGAG	CACCAGCTGGTTATCTCTCAGCTC	354	NM_000594.4
*TLR-2*	GCCCATTGCTCTTTCACTGCTT	ATGACCCCCAAGACCCACAC	96	NM_003264.4
*TLR-4*	CCCGACAACCTCCCCTTCTC	GGGCTAAACTCTGGATGGGGT	211	NM_003266
*UL83*	GCAGCCACGGGATCGTACT	GGCTTTTACCTCACACGAGCATT	159	NC_006273
*β-actin*	ACT GGA ACG GTG AAG GGT GAC	AGA GAA GTG GGG TGG CTT TT	169	NM_001101

## Abbreviations

ASCs	Adipose-derived MSCs
ATRA	All-trans retinoic acid
CK	Cytokeratin
CPE	Viral load dependent cytopathological effect
FBS	Fetal bovine serum
HCMV	Human cytomegalovirus
ICAM-1	Intercellular adhesion molecule 1
IFNγ	γ−Interferon
IL	Interleukin
MCP-1	Monocyte chemotactic protein
MSCs	Mesenchymal stromal/stem cells
MOI	Multiplicity of infection
PCR	Polymerase chain reaction
prASCs	ASCs from perirenal adipose tissue
qPCR	Quantitative real-time polymerase chain reaction
RT	Room temperature
TLR	Toll-like receptor
TNFα	Tumor necrosis factor-α
UL83	HCMV-specific mRNA of UL83-coded phosphoprotein 65
